# Glycated Albumin Guided Therapy in Newly Diagnosed Type 2 Diabetes A Randomized Controlled Trial

**DOI:** 10.1111/1753-0407.70263

**Published:** 2026-08-17

**Authors:** Qian Ren, Rui Zhang, Wei Huang, Xiaojie Yang, Jian Xu, Yufeng Li, Shanshan Lin, Junting Duan, Huixian Yan, Fang Chen, Zhihui Wang, Hongyu Liu, Jingjing Wang, Xiaoya Chen, Ling Chen, Ying Gao, Ping Zhang, Tianhao Ba, Kohzuma Takuji, Linong Ji

**Affiliations:** ^1^ Department of Endocrinology Peking University People's Hospital Beijing China; ^2^ Department of Endocrinology Beijing Haidian Hospital Beijing China; ^3^ Department of Endocrinology The Third People's Hospital of Datong City Datong Shanxi China; ^4^ Department of Endocrinology, Beijing Tiantan Hospital Capital Medical University Beijing China; ^5^ Department of Endocrinology Beijing Pinggu Hospital Beijing China; ^6^ Department of Endocrinology Beijing Shijingshan Hospital Beijing China; ^7^ Department of Endocrinology Civil Aviation General Hospital Beijing China; ^8^ Diagnostics Division Asahi Kasei Pharma Corporation Tokyo Japan; ^9^ Peking University Diabetes Center Bejing China

**Keywords:** combination therapy, early initiation, glycated albumin, newly diagnosed type 2 diabetes mellitus

## Abstract

**Background:**

Glycated albumin (GA) has already been introduced in clinic for decades. However, it remains unclear whether routine measurement of GA could serve as a practical tool to improve the care of patients with type 2 diabetes (T2DM).

**Methods:**

This multicenter, randomized controlled trial enrolled 200 patients with newly diagnosed, poorly controlled type 2 diabetes from seven sites in China. Participants were randomly assigned (1:1) to either GA‐guided therapy (treatment intensification if GA > 16% at 4 weeks) or standard therapy, with a 12‐weeks follow‐up. The primary outcome was the proportion of patients achieving HbA1c < 7%; secondary outcomes included HbA1c < 6.5%, glycemic parameters, hypoglycemia, and β‐cell function.

**Results:**

A total of 200 patients were included in this study. Therapy compliances were 97.4% (SD, 5.3%) and 98.0% (SD, 8.2%) in the GA‐guided therapy group and standard therapy group, respectively. There was no significant difference in the achievement rate of HbA1c (< 7%) between the GA‐guided therapy group (64.3%) and the standard therapy group (64.4%) after the 12‐week follow‐up (*p* = 0.99). In the secondary outcome, the achievement rate of HbA1c (< 6.5%) was higher in the GA‐guided therapy group than in the standard therapy group (40.5% vs. 25.3%, *p* = 0.034). Furthermore, the changes in HbA1c, HOMA‐β, HOMA‐IR, body weight reduction were all better in the GA guided therapy group than in standard therapy group.

**Conclusion:**

GA‐guided therapy did not enhance HbA1c < 7% achievement; however, its potential benefits for more stringent glycemic targets and metabolic parameters in early diabetes management deserve further investigation.

**Trial Registration:** ClinicalTrials.gov: NCT05227677

## Highlights

- GA‐guided therapy is an actionable tool in early initiation of combination therapy.
- The potential of GA‐guided therapy to improve metabolic outcomes in early diabetes management merits further investigation.

## Graphical abstract

Early initiation of combination therapy guided by GA may have potential benefit in further reduction in HbA1c, improvement in HOMA β and HOMA‐IR, without increasing the risk of hypoglycemia.

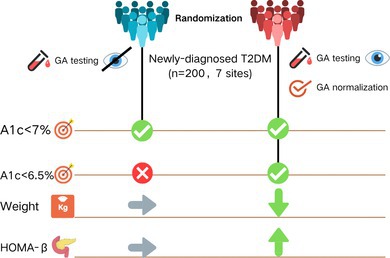

## 1 Introduction

HbA1c is a gold standard and a clinically actionable biomarker that is widely used in clinical practice and trials [1, 2, 3]. However, this indicator is often insensitive to short‐term medication adjustments or intensification. In comparison, Glycated albumin (GA) reflects blood glucose levels within the past month because the half‐life of albumin is shorter than that of hemoglobin [4]. The American Diabetes Association (ADA)'s 2024 guidelines have recommended GA for clinical use as an alternative measure of glycemia in people with diabetes [2]. Moreever, a recent study in 2025 further provided compelling evidence for the concordance between GA and short‐term hyperglycemia metrics from continuous glucose monitoring (CGM) in type 2 diabetes patients [5].

However, most clinicians remain uncertain about how to appropriately use this biomarker. Furthermore, it is still unclear whether treatment guidance based on GA can lead to improved metabolic outcomes. This current challenge in the clinical application of GA mirrors that faced over 30 years ago when HbA1c was first introduced into clinical practice. At that time, although physicians understood its clinical significance, it was unclear whether adjusting treatment based on HbA1c would lead to improved patient outcomes. It was not until 1990 that a randomized controlled trial (RCT) demonstrated for the first time the clinical importance of using HbA1c to guide treatment adjustments [1]. Clinical trials may pave the way for transforming a “useful” biomarker into a “practical” one in clinical practice.

GA has already been introduced in clinic for decades. Consequently, we contend that there is currently a need for a clinical trial to validate GA as a “practical” biomarker. The hypothesis of this study is that GA‐guided therapy is an actionable and beneficial strategy for the treatment of patients with newly‐diagnosed type 2 diabetes.

## 2 Research Design and Methods

### 2.1 Study Design and Participants

This multicenter, randomized controlled trial was conducted in seven hospitals in China (ClinicalTrials.gov NCT05227677; Table S1). Eligible patients were 20–70 years of age; newly diagnosed T2DM or duration of T2DM less than 1 year; without antidiabetic medications for 3 months prior to screening; and HbA1c ≥ 7.5% and < 10.5%. The main exclusion criteria were as follows: severe diabetic chronic or acute complications in the 3 months prior to screening; severe comorbidities of major organs; thalassemia, haemolytic anemia, and other diseases that may cause erythrocyte instability and affect the measurement of HbA1c; treatment with calcium dobesilate or a disease requiring calcium dobesilate treatment (calcium dobesilate may affect the measurement of GA); two or more episodes of severe hypoglycaemia in the recent 1 year prior to screening; and patients who needed to initiate treatment with sulfonylureas, glinides, insulin, or insulin analogues as evaluated by investigators (the criterion was set to minimize potential hypoglycemia during intensive GA‐normalizing combination therapy). The full exclusion criteria are listed in Table S2 and ClinicalTrials.gov, NCT05227677.

### 2.2 Randomization

Patients newly diagnosed with T2DM were 1:1 randomly assigned to one of two groups: GA‐guided therapy or standard therapy. A block randomization method was used to maintain the balance between the two groups. Block randomization (1:1 ratio, block size multiples of 2) was generated using SAS 9.4, with 400 preassigned codes (001–400) to ensure flexibility. Sealed randomization envelopes, prepared by an independent statistician, maintain allocation concealment. Clinical study personnel remain blinded to the subject randomization allocation table.

### 2.3 Procedure

After obtaining informed consent, patients with newly diagnosed T2DM were 1:1 randomly assigned to the GA‐guided or standard therapy groups, as shown in Figure S1. At baseline, A1c and other metabolic indices (excluding GA) were measured. All patients were treated with antidiabetic therapy according to the Guidelines for the Prevention and Treatment of Type 2 Diabetes in China (2020) [6] and were followed up for 12 weeks at 4‐week intervals in both groups. The samples for GA testing were collected at 4‐week intervals. In Week 4, in the GA‐guided therapy group, the antidiabetic treatment regimen was strengthened when the GA value was higher than 16% at the first 4 weeks, following the “GA normalization strategy” (The normal reference range for GA is 11%–16%). If GA ≤ 16%, no adjustment is made unless hypoglycemia is reported. In the standard therapy group, investigators were unaware of the GA value and relied on current guidelines to adjust the treatment. In Week 8, no treatment adjustments unless medication‐related AEs occur. Unexplained hypoglycemia will prompt dose reductions if necessary. After 12 weeks, A1c and other metabolic indices, including GA, were measured. All antidiabetic drugs were permitted in this study except sulfonylureas, glinides, insulin, and insulin analogues. This study was approved by the ethics committees of all study sites, and informed consent was obtained from all participants. This study was conducted in compliance with the clinical trial protocol and ethical principles of the Declaration of Helsinki.

### 2.4 GA Masking

In the standard therapy group, serum samples for GA testing were collected at baseline and at Week‐4 and Week‐8 and stored at −80°C freezers in all the sites to ensure that patients and investigators were unaware of GA levels during the study, and that therapy in the standard therapy group was not influenced by GA. All sites maintained temperature monitoring records for stored GA specimens, with all readings complying with prespecified requirements. Site‐initiated GA assays were deferred until after the last patient last visit (LPLV) for the above samples in accordance with the blinded study design.

In the GA‐guided therapy group, site‐based GA quantification was performed immediately post‐collection in serum samples collected at Week 4 in the GA‐guided therapy group. At the final visit (Week 12), since GA measurement no longer affected treatment adjustments, GA levels were assayed on the day of blood collection in both groups.

### 2.5 Biochemical Testing of GA

Serum GA levels were measured using an enzymatic method (Lucia GA‐L; Asahi Kasei Pharma Corp., Tokyo, Japan) according to the manufacturer's instructions in each site. Regarding GA measurement performance, the overall repeatability (%CV) ranged from 0.4% to 3.7% for GA values (%), GA concentrations, and albumin concentrations, while the within‐laboratory precision (%CV) for these measurements ranged from 0.8% to 4.2% [7]. The GA value showed good linearity from 9.4% to 54.9% across the assay range (*y* = 0.993*x* + 2.880; *R* ^2^ = 0.9998) with a good recovery from 98.9% to 100.9% [7]. The above data indicate that GA measurement has sufficiently low inter‐assay and intra‐assay CV.

### 2.6 Sample Size Calculation

The sample size calculation was based on a pilot single‐center study conducted in our hospital. The sample size was 40 for the pilot study; 68% were men, with average age 52 ± 14.5 years old, BMI 27.8 ± 3.9 kg/m^2^, and baseline HbA1c 9.1% ± 1.5%. The results of this pilot study showed that 76.9% of patients who adjusted their treatment plan according to GA achieved HbA1c < 7% after 3 months of follow‐up, whereas the control group had a rate of 46.2% (*p* = 0.226). We started our calculations by taking *p* < 0.05 as a statistically significant difference and 0.8 as the statistical power, with a difference of 30% between the experimental group and the control group as the calculation basis. Therefore, the calculated sample size is 39 cases per group. The target sample size for the GA‐guided therapy group and standard therapy group in this study was determined to be 100 individuals. We exceeded the calculated sample size (*N* = 78) by recruiting 200 participants due to the following reasons: Accounting for potential data variability in real‐world settings, improving the robustness of secondary endpoint assessments, and availability of additional funding.

### 2.7 Evaluation of Compliance

Compliance (%) = The actual total dose (mg)/Prescribed total dose (mg) × 100%, with the result rounded to one decimal place. Good compliance was defined as taking 80%–120% of the drug dose during this study.

The actual total dose (mg) = Prescribed total dose (mg) + Excessive total dose (mg) if participants took more than the prescribed dose.

The actual total dose (mg) = Prescribed total dose (mg) − Missed total dose (mg) in the event of missed doses.

Prescribed total dose (mg) = $\sum_{i=3A/3B}^{5}$ Single dose mg × dosing frequency × next prescription start date / actual date of Visit 5 −current prescription start date; where for once‐daily dosing, dosing frequency = 1; for twice‐daily dosing, dosing frequency = 2; For three‐times‐daily dosing, dosing frequency = 3; for four‐times‐daily dosing, dosing frequency = 4; for every‐other‐day dosing, dosing frequency = 0.5; for once‐weekly dosing, dosing frequency = 1/7; *i* for Visits 3A/3B, 4, and 5.

Excessive/missed total dose (mg) = $\sum_{i=3A/3B}^{5}$ total dose of excessive / missed mg since the previous visit, where *i* for Visits 3A/3B, 4, and 5.

### 2.8 Outcomes

The primary outcome was the achievement rate of HbA1c (< 7%) at the 12‐week follow‐up. The secondary outcomes were as follows: the achievement rate of HbA1c (< 6.5%), the extent of change of HbA1c, incidence and frequency of hypoglycaemia, the changes of islet β‐cell function (HOMA‐β), the changes of insulin resistance (HOMA‐IR), and the changes of body weight, waist circumference and BMI between the two groups at 12 weeks of follow‐up. HOMA‐β and HOMA‐IR were calculated as described previously [8].

### 2.9 Statistical Methods

Quantitative parameters were presented as mean, standard deviation (SD), or median (interquartile range), as appropriate. The description of the classification parameters is based on numbers and percentages. The primary outcome was analyzed using the chi‐square test. 95% confidence intervals (95% CIs) were calculated using Wald's method. For the secondary outcome, Student's *t*‐test was used to compare normally distributed quantitative variables between the groups. The Wilcoxon rank‐sum test was used to compare non‐normally distributed quantitative variables between groups, and categorical variables were compared between groups using the chi‐square test. All statistical tests were conducted using a two‐sided test, and differences with a *p* value less than 0.05 were considered statistically significant.

The analysis included three datasets. The full analysis set (FAS) was defined as participants who were randomized, met the study inclusion criteria, or had baseline measurements of the primary efficacy endpoint. The per‐protocol set (PPS) was defined as subjects with good compliance to the FAS and no significant protocol deviations that affected the primary efficacy evaluation. The safety set (SS) was defined as all randomized subjects who received at least one treatment and underwent a safety evaluation. The primary and secondary outcomes were analyzed using FAS and PPS. The safety outcomes were analyzed using SS, and all analyses were performed using SAS statistical analysis software version 9.4.

## 3 Results

The study was conducted between August 2022 and August 2024. A total of 207 patients with T2DM provided informed consent to participate in this study, and 200 patients were randomized into the GA‐guided therapy group (*n* = 98) or the standard therapy group (*n* = 102). A total of 199 (99.5%) patients were included in the FAS (one patient from the NC group was excluded because of a violation of the inclusion criteria for newly diagnosed diabetes). A total of 179 (89.5%) participants completed the study (Figure S2), and 21 patients withdrew from this study. Voluntary termination was the most common reason (81.8%).

The baseline clinical characteristics of all patients are shown in Table 1. In brief, the average age [49.3 (10.8) and 50.7 (10.7) years old], HbA1c [8.9 (0.9) and 8.7 (0.8)%], GA [22.5 (4.1) and 21.9 (3.5)%] were all similar between the GA‐guided therapy group and the standard therapy group.

**TABLE 1:** Clinical characteristics of the patients at baseline.

Characteristics	GA‐guided therapy group	Standard therapy group
No.	98	101
Male (%)	66 (67.3)	65 (64.4)
Age (years)	49.3 (10.8)	50.7 (10.7)
BMI (kg/m^2^)	28.2 (3.5)	27.7 (4.5)
Waist circumference (cm)	97.0 (8.4)	94.7 (11.2)
Systolic blood pressure (mmHg)	135 (17)	133 (13)
Diastolic blood pressure (mmHg)	86 (11)	85 (11)
Hyperlipidemia (%)	57 (58.2)	55 (54.5)
Hypertension (%)	36 (36.7)	41 (40.6)
FBG (mmol/L)	9.93 (1.93)	9.31 (1.83)
HbA1c (%)	8.9 (0.9)	8.7 (0.8)
GA (%)	22.5 (4.1)	21.9 (3.5)
Fasting serum insulin (μU/mL)	11.73 (7.99–17.40)	11.55 (8.32–18.50)
Fasting serum C peptide (ng/mL)	2.91 (2.26–3.71)	2.88 (2.16–3.51)
HOMA‐IR	5.30 (3.50–7.40)	4.80 (3.30–7.90)
HOMA‐β	37.6 (24.5–63.6)	43.7 (25.9–75.6)
Leukocyte (×10^9^/L)	6.65 (1.46)	7.18 (1.97)
Erythrocyte (×10^12^/L)	5.02 (0.44)	5.06 (0.52)
Platelet (×10^9^/L)	245 (63.4)	241 (59.0)
Hemoglobin (g/L)	153.5 (16.02)	153.8 (14.26)
Serum albumin (g/L)	46.62 (2.82)	46.46 (2.72)
ALT (mmol/L)	33.3 (21.2–49.0)	35.4 (22.4–50.1)
Serum Creatinine (μmol/L)	66.5 (14.1)	65.2 (15.0)
eGFR (mL/mim/1.73 m^2^)	106.3 (18.1)	106.0 (18.3)
Serum uric acid (mmol/L)	348.4 (277.0–398.1)	323.0 (281.0–370.0)
Total cholesterol (mmol/L)	5.36 (1.11)	5.17 (0.99)
Triglyceride (mmol/L)	2.08 (1.48–2.85)	1.92 (1.38–3.15)
HDL‐C (mmol/L)	1.14 (0.25)	1.18 (0.26)
LDL‐C (mmol/L)	3.43 (1.00)	3.25 (0.86)
Concomitant medical conditions (%)		
Metabolism and nutritional disorders	79 (80.6)	75 (74.3)
Vascular discorders	36 (36.7)	41 (40.6)
Hepatobiliary system disorders	34 (34.7)	29 (28.7)
Concomitant medications (%)		
Lipid‐modifying drugs	42 (42.9)	44 (43.6)
RAS‐acting agents	24 (24.5)	23 (22.8)
Calcium channel blockers	14 (14.3)	23 (22.8)
β‐blockers	10 (10.2)	8 (7.9)
Diuretics	3 (3.1)	3 (3.0)

*Note:* Data are *n* (%), mean (SD), or median (interquartile range) in FAS.

Abbreviations: ALT, alanine aminotransferase; BMI, body mass index; eGFR, estimated glomerular filtration rate; HDL‐C, high‐density lipoprotein cholesterol; HOMA‐β, homeostatic model assessment for β‐cell function; HOMA‐IR, homeostatic model assessment for insulin resistance; LDL‐C, low‐density lipoprotein cholesterol; RAS, renin–angiotensin system.

All patients were prescribed antidiabetic drugs according to the Guidelines for the Prevention and Treatment of Type 2 Diabetes Mellitus in China (2020) [6]. The top three initial hypoglycaemic drugs were metformin (90.8% vs. 90.1%), sodium‐dependent glucose transporter 2 inhibitors (SGLT2i) (9.2% vs. 11.9%), and dipeptidyl peptidase 4 inhibitors (DPP‐4i) (5.1% vs. 5.0%) at baseline in the GA‐guided and standard therapy groups, respectively. The percentages of monotherapy and combination therapy at baseline and during follow‐up are shown in Figure S3. After adjustment for treatment according to the GA‐guided principle, the proportion of monotherapy decreased from 76.5% to 36.3% in the GA‐guided therapy group, whereas in the standard therapy group, the proportion of monotherapy varied between 82.2% at baseline and 81.1% after 12 weeks of follow‐up. The detailed changes of therapy, GA and HbA1c levels in each visit was shown in Table S3. Therapy compliances were 97.4 (5.3) and 98.0 (8.2)% in the GA guided therapy group and standard therapy group, respectively. The changes of GA from baseline were shown in Figure S4.

**FIGURE 1:**
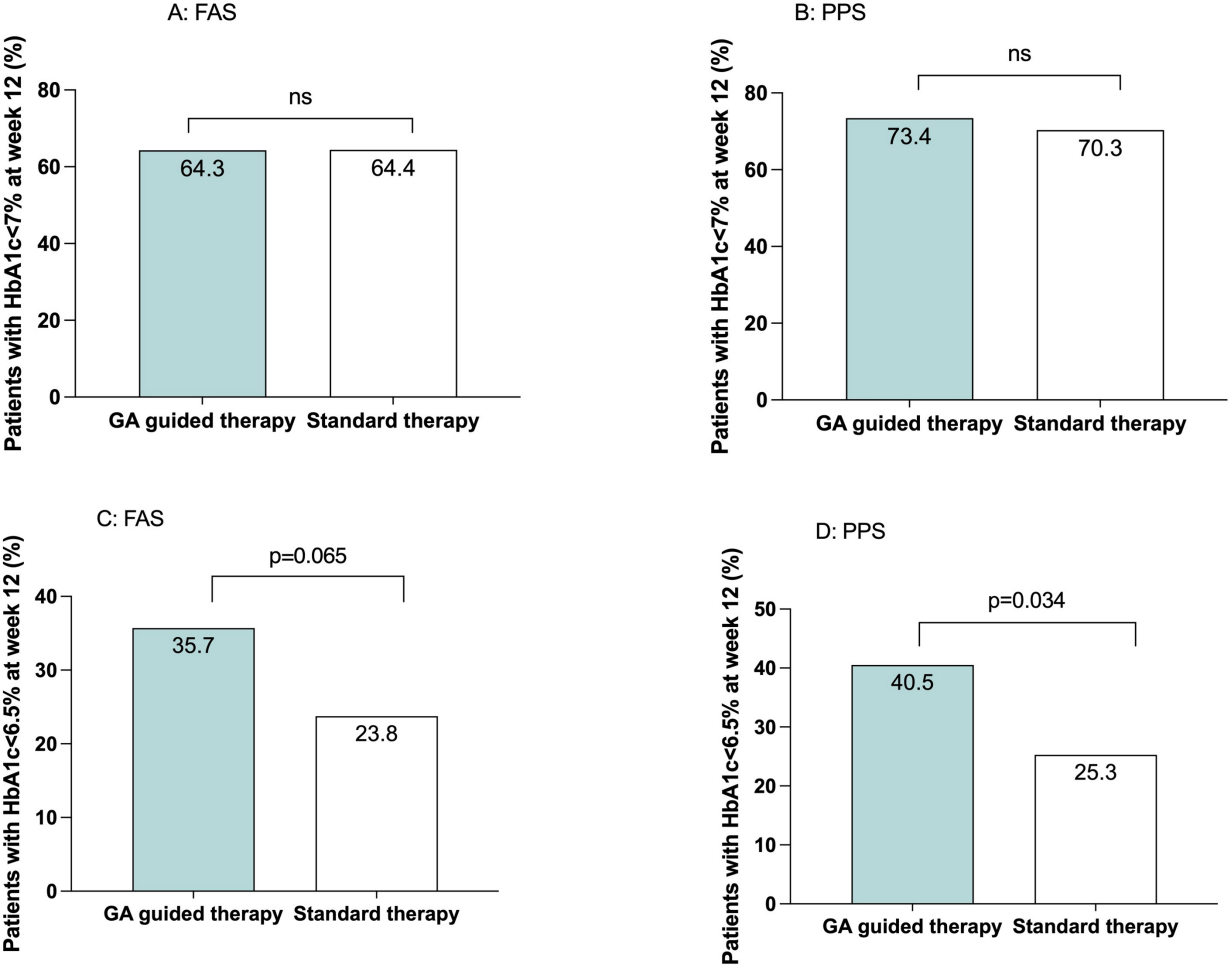
The achievement of HbA1c between the two groups (A: the achievement of HbA1c < 7% in FAS; B: the achievement of HbA1c < 7% in PPS; C: the achievement of HbA1c < 6.5% in FAS; D: the achievement of HbA1c < 6.5% in PPS).

The chi‐square test showed no significant difference in the achievement rate of HbA1c (< 7%) between the GA guided therapy group (64.3%, 95% CI 54.8%–73.8%) and the standard therapy group (64.4%, 95% CI 55.0%–73.7%) after the 12‐week follow‐up (−0.07%, 95% CI −13.4 to 13.2%, *p* = 0.99), as shown in Figure 1A. This result was similar for the PPS (Figure 1B).

**FIGURE 2:**
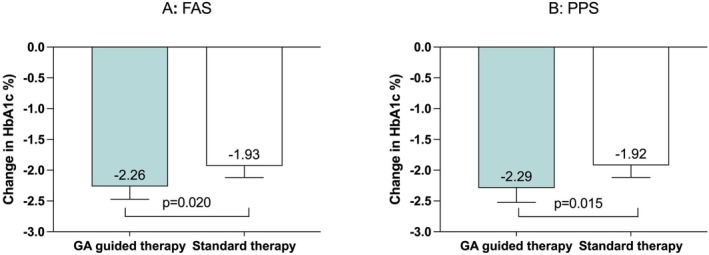
The change in HbA1c between the two groups (A: the change in HbA1c in FAS; B: the change in HbA1c in PPS).

The analysis of secondary outcomes showed a marginal difference in the achievement rate of HbA1c (< 6.5%) between the GA‐guided therapy group (35.7%) and the standard therapy group (23.8%) after the 12‐week follow‐up (*p* = 0.065) in the FAS analysis (Figure 1C). The achievement rate of HbA1c (< 6.5%) was higher in the GA‐guided therapy group than in the standard therapy group (40.5% vs. 25.3%, *p* = 0.034) (Figure 1D) in the PPS analysis. We then compared the changes in HbA1c levels and found that the change in HbA1c from baseline to Week 12 was significantly better in the GA‐guided therapy group [−2.26 (1.03)%] than in the standard therapy group [−1.93 (0.96)%] (*p* = 0.020) in FAS analysis, as shown in Figure 2A. A similar result was noted in the PPS analysis: the change in HbA1c from baseline to Week 12 was −2.29 (1.06)% and −1.92 (0.95)% in the GA‐guided therapy group and standard therapy group, respectively (*p* = 0.015) (Figure 2B).

**FIGURE 3:**
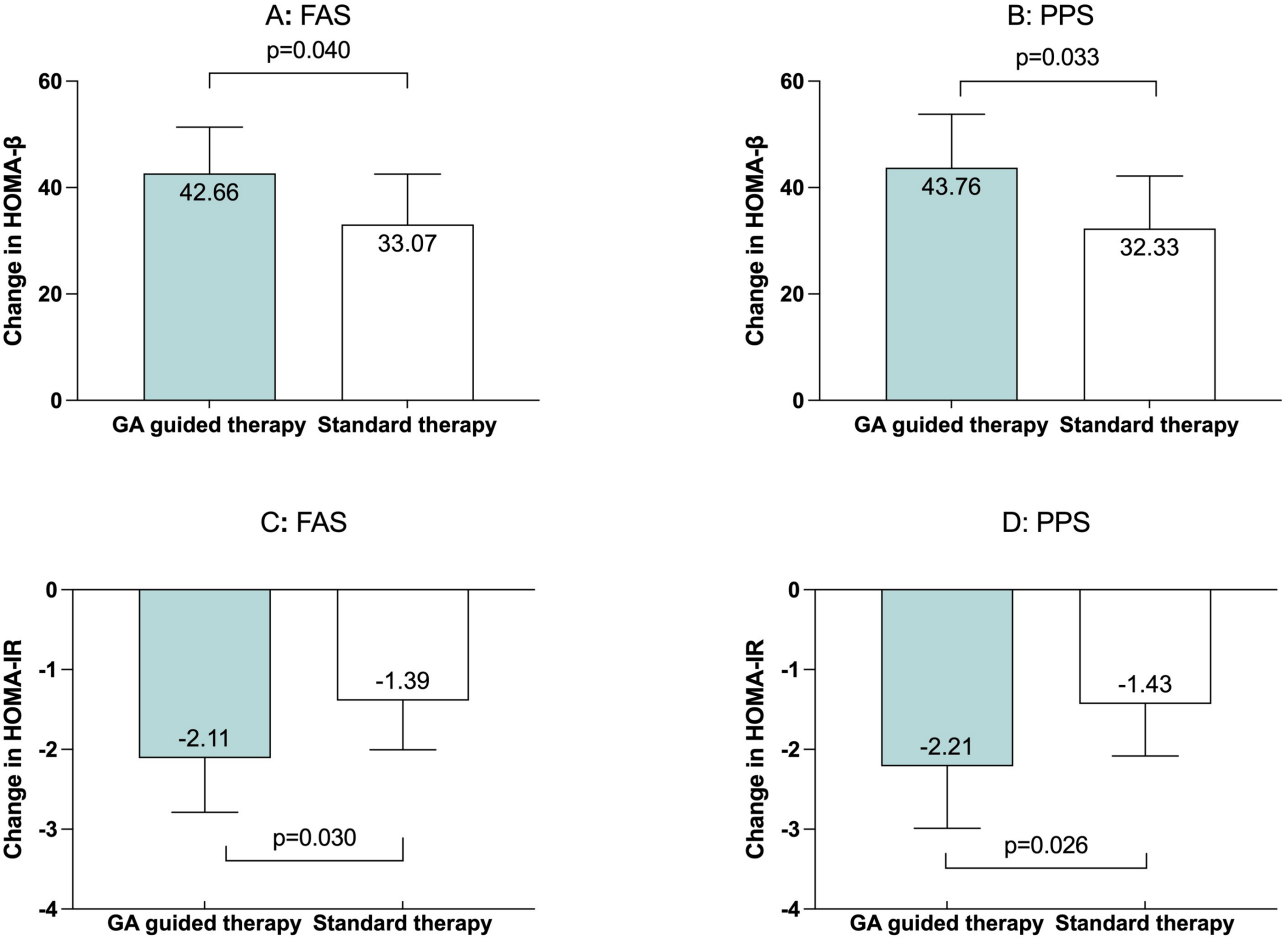
The changes in islet β cell function and insulin resistance between the two groups (A: the change in HOMA‐β in FAS; B: the change in HOMA‐β in PPS; C: the change in HOMA‐IR in FAS; D: the change in HOMA‐IR in PPS).

Regarding the effect of GA‐guided therapy on islet β‐cell function and insulin resistance, we found that the improvement of HOMA‐β was significantly better in the GA‐guided therapy group than in the standard therapy group (*p* = 0.040 in FAS and *p* = 0.033 in PPS). The alleviation of insulin resistance (HOMA‐IR) was also significantly better in the GA‐guided therapy group than in the standard therapy group (*p* = 0.030 in FAS and *p* = 0.026 in PPS), as shown in Figure 3A–D.

In this study, sulphonylureas, glinides, insulin, and insulin analogues were forbidden. In the entire study population, body weight, BMI, and waist circumference decreased after the antidiabetic treatment. And more impressively, we found the reduction in body weight (−3.03 vs. −1.72 kg, *p* = 0.009), BMI (−1.08 vs. −0.63 kg/m^2^, *p* = 0.009), and waist circumference (−4.14 vs. −2.39 cm, *p* = 0.006) were more prominent in GA guided therapy group than in the standard therapy group in the FAS analysis. Similar results were observed in the PPS analysis (Figure S5A–F).

Safety analyses revealed that adverse events occurred in 32 (32.7%) and 26 (25.5%) patients in the GA‐guided and standard therapy groups, respectively. More importantly, the adverse event of special interest (AESI) was hypoglycaemia, and the risk of hypoglycaemia was low in this study (2.5%). In total, three patients reported mild hypoglycaemia during follow‐up (two in the GA‐guided therapy group and one in the standard therapy group).

## 4 Discussion

In this randomized controlled trial where investigators and patients were blinded to GA results in the control group, adjustment of treatment based on GA did not compromise the attainment of HbA1c < 7% in patients with newly diagnosed type 2 diabetes. Analysis of secondary outcomes indicated that GA‐guided early combination therapy was associated with greater HbA1c reduction and potential improvements in pancreatic β‐cell function, insulin resistance, and body weight, without an apparent increase in hypoglycemia risk.

Our trial design was informed by the historic study that first established the clinical utility of HbA1c. In that pioneering RCT, the intervention group had HbA1c measured quarterly, while the control group received care without this information. The demonstration that HbA1c monitoring improved metabolic control by guiding therapy changes provided a direct model for our study, applying the same principle to evaluate GA [1].

While our trial design pays homage to the classic A1c‐guided therapy RCTs from three decades ago in its grouping and blinding methodology, a key distinction lies in our implementation of a prespecified treatment protocol targeting “GA normalization.” We propose the “GA normalization” strategy for two primary reasons. First, in adjusting blood glucose regimens, both clinicians and patients require an “actionable tool” that can sensitively reflect short‐term therapeutic efficacy. Such a tool enables faster and more precise regimen optimization, thereby helping to overcome clinical inertia. GA serves this purpose well, as changes in its levels have been shown to predict subsequent HbA1c changes after 3 months [9]; underscoring its utility as a clinical biomarker for diabetes [4, 10, 11]. Another rationale for the “GA normalization” strategy is the recent advent of numerous antidiabetic agents with a low risk of hypoglycemia [12]. The favorable risk profile of these drugs provides a safer foundation for clinicians to pursue more intensive glycemic targets. We therefore contend that the current therapeutic landscape is opportune for introducing the concept of “GA normalization.”

After 12 weeks of follow‐up, introduction and drug adjustment based on GA did not show an advantage for common glycated hemoglobin target values (< 7%). Thus, the primary outcome of this study was a negative result. A possible explanation for this is that the target value of HbA1c < 7% may be relatively easy to achieve in newly diagnosed and initially treated patients. We have also observed in other clinical trials that the reduction of glycated hemoglobin by monotherapy in drug‐naïve patients is generally better than the drug‐lowering effect of metformin combination therapy [13]. Notably, when targeting a more stringent HbA1c threshold of 6.5%, preliminary signals of GA's clinical value began to emerge. In addition, in the secondary outcome of HbA1c reduction, GA‐guided therapy was better than standard therapy. Therefore, we hypothesized that the advantage of GA lies in the guidance of tight antidiabetic treatments.

Another noteworthy finding from this study was that in the GA‐guided therapy group, although the percentage of patients with HbA1c < 7% was similar to that of the standard care group, there were better improvements in islet β‐cell function, insulin resistance, and associated metabolic parameters. The higher combination therapy rates in the GA‐guided therapy group may explain this. Furthermore, the proportion of patients receiving combination therapy increased from 23.5% to 63.7% in the GA‐guided therapy group, whereas in the standard therapy group, the proportion of combination therapy varied between 17.8% at baseline and 18.9% after 12 weeks of follow‐up. Type 2 diabetes mellitus is heterogeneous, and combination therapy may better focus on various aspects of the pathophysiological mechanism of type 2 diabetes (ominous octet) [14], as shown in the previous EDICT study [15, 16] and VERIFY study [17]. Although comparable HbA1c < 7% achievement rates were found between groups, our analysis of secondary outcomes suggested a potential trend toward enhanced metabolic improvement with GA‐guided therapy.

This study has three notable strengths. First, this was the first multicenter, randomized, interventional trial to use GA as an index. Second, we proposed a new idea of “GA normalization” in exploring treatment strategies in diabetes, and our findings indicate that GA is an effective strategy for facilitating earlier combination therapy without increasing hypoglycemic risk. Third, our analysis of secondary outcomes indicated that the GA‐guided group may have experienced some advantages, including modestly greater HbA1c reductions, higher rates of HbA1c < 6.5%, and better improvements in β‐cell function and metabolic profiles. However, these findings should be interpreted with caution given the secondary nature of these endpoints.

This study had some limitations that should be noted when interpreting our findings. First, we only included patients who were newly diagnosed with diabetes. For patients with diabetes with a long disease duration, whether GA has similar treatment guiding significance requires further exploration in the future. Second, the use of long‐acting injectable drugs, such as GLP‐1RA, was present in a relatively small proportion of patients in this study. Therefore, the clinical value of GA in injectable drug treatments requires further investigation. Third, this study had a slightly higher dropout rate of 10.5%. However, the study protocol proactively addressed this by enrolling a sample size 2.56 times larger than the statistically estimated requirement, thereby mitigating the potential impact of attrition on outcome assessments. Forth, the present study was conducted in a Chinese population; to enhance the generalizability of the findings, it would be preferable to conduct a similar study in people from other countries and ethnic groups.

## 5 Conclusion

This study concluded that while GA‐guided therapy did not increase the rate of achieving HbA1c < 7%, it emerges as an actionable tool for optimizing key metabolic parameters. These promising findings warrant further evaluation.

## Author Contributions

Q.R. wrote the manuscript. L.J. and Q.R. designed the study and reviewed and edited the manuscript. R.Z., W.H., X.Y., J.X., Y.L., S.L., J.D., H.Y., F.C., Z.W., H.L., J.W., X.C., L.C., Y.G., and P.Z. contributed to data acquisition. R.Z., Q.R., and T.B. analyzed and interpreted the data. K.T. and L.J. contributed to the discussion and reviewed and edited the manuscript. L.J. is the guarantor of this work and, as such, had full access to all the data in the study and takes responsibility for the integrity of the data and the accuracy of the data analysis. All the authors have read and approved the final manuscript and had full access to all the data in the study and accept responsibility to submit for publication.

## Funding

This work was supported by Asahi Kasei Pharma Corporation and National Clinical Key Specialty Construction Program of China (Department of Endocrinology, Peking University People's Hospital) with support from the central government budget.

## Conflicts of Interest

K.T. is an employee of Asahi Kasei Pharma Corporation. The other authors declare no conflicts of interest.

## Supporting information


**Table S1:** List of study sites.
**Table S2:** The full exclusion criteria.
**Table S3:** Changes of therapy, HbA1c and GA during follow up.
**Figure S1:** Study design.
**Figure S2:** Enrollment, randomization, and follow up of study participants.
**Figure S3:** Changes in the proportion of monotherapy and combination therapy during follow up (A: GA guided therapy group; B: standard therapy group).
**Figure S4:** Changes in GA during follow up.
**Figure S5:** The changes in metabolic paramaters between the two groups (A: the change in body weight in FAS; B: the change in body weight in PPS; C: the change in BMI in FAS; D: the change in BMI in PPS; E: the change in waist circumference in FAS; F: the change in waist circumference in PPS).

## Data Availability

The data that support the findings of this study are available on request from the corresponding author. The data are not publicly available due to privacy or ethical restrictions.
