# Supplementary material for: Glycated Albumin Guided Therapy in Newly Diagnosed Type 2 Diabetes A Randomized Controlled Trial
Source: J Diabetes. 2026 Aug 17;18(8):e70263. doi: 10.1111/1753-0407.70263 (PMC13481313; doi:10.1111/1753-0407.70263)
Supplement: Supplementary file 1 [file JDB-18-e70263-s001.docx]

**Supplementary materials**

Table S1 List of study sites----------------------------------------------------2

Table S2 The full exclusion criteria--------------------------------------------- 2

Table S3 Changes of therapy, HbA1c and GA during follow up---------------------------------- 3

Figure S1：Study design-----------------------------------------------------3

Figure S2: Enrollment, randomization and follow up of study participants------------3

Figure S3: Changes in the proportion of monotherapy and combination therapy during follow up------------------------------------------------------------------4

Figure S4: Changes in GA during follow up--------------------------------------4

Figure S5: The changes in metabolic parameters between the two groups-----------5

**Table S1** List of study sites

| No. | Sites |
| --- | --- |
| 1 | Peking University People's Hospital |
| 2 | Beijing Haidian Hospital |
| 3 | The Third People's Hospital of Datong |
| 4 | Beijing Tiantan Hospital |
| 5 | Beijing Pinggu Hospital |
| 6 | Beijing Shijingshan Hospital |
| 7 | Civil Aviation General Hospital |

**Table S2** The full exclusion criteria

| No. | Exclusion criteria |
| --- | --- |
| 1 | Requiring treatment of proliferative retinopathy, maculopathy, painful diabetic neuropathy, diabetic foot, diabetic ketoacidosis, or hyperglycemic hyperosmolar status in recent 3 months prior to screening |
| 2 | The following history or conditions in recent 6 months prior to screening: (i) decompensated cardiac insufficiency (NYHA class III or IV); (ii)myocardial infarction, coronary artery bypass grafting or coronary stent implantation; (iii) uncontrolled severe arrhythmia and is not suitable to participate in this study evaluated by the investigator; (iv) hemorrhagic stroke or ischemic stroke and is not suitable to participate in this study evaluated by the investigator. |
| 3 | Laboratory indicators meet one of the following criteria: (i) alanine aminotransferase (ALT) or aspartate aminotransferase (AST) > 3× upper limit of normal(ULN); (ii) total bilirubin > 2× ULN; (iii) hemoglobin <100g/L; (iv)total protein <60g/L; (v)albumin <30g/L; (vi) glomerular filtration rate(eGFR)<60 ml min-1 per 1.73 m^2^. |
| 4 | Thalassemia, hemolytic anemia and other diseases that may cause erythrocyte instability and affect the measurement of HbA1c |
| 5 | Uncontrolled thyroid dysfunction |
| 6 | Systemic corticosteroids treatment in recent 1 month prior to screening (exception of inhalation or local external treatment) |
| 7 | Pregnancy or lactating |
| 8 | Two or more episodes of severe hypoglycemia in recent 1 year prior to screening |
| 9 | Patient who needs to initiate treatment with sulfonylureas, glinides, insulin or insulin analogues evaluated by the investigators |
| 10 | Patient is being treated with calcium dobesilate or has a disease requiring calcium dobesilate treatment, or may be treated with calcium dobesilate in the near future evaluated by the investigators |
| 11 | Patients considered unsuitable for observation by investigators |

**Table S3** Changes of therapy, HbA1c and GA during follow up

|  | GA-guided therapy group | | | | Standard therapy group | | | |
| --- | --- | --- | --- | --- | --- | --- | --- | --- |
| Visit (week) | 0 | 4 | 8 | 12 | 0 | 4 | 8 | 12 |
| No. | 98 | 91 | 91 | 86 | 101 | 95 | 95 | 93 |
| Metformin | 89 (90.8) | 83 (91.2) | 82 (90.1) | / | 91 (90.1) | 86 (90.5) | 85 (89.5) | / |
| SGLT2 inhibitors | 9 (9.2) | 22 (24.2) | 24 (26.4) | / | 12 (11.9) | 11 (11.6) | 13 (13.7) | / |
| DPP-4 inhibitors | 5 (5.1) | 27 (29.7) | 27 (29.7) | / | 5 (5.0) | 7 (7.4) | 5 (5.3) | / |
| α-glucosidase inhibitors | 5 (5.1) | 10 (11.0) | 10 (11.0) | / | 4 (4.0) | 4 (4.2) | 4 (4.2) | / |
| Glitazones | 0 | 0 | 0 | / | 0 | 1 (1.1) | 0 | / |
| Oral fixed-dose combination hypoglycemic drugs | 7 (7.1) | 7 (7.7) | 7 (7.7) | / | 2 (2.0) | 2 (2.0) | 2 (2.1) | / |
| GLP-1 receptor agonists | 1 (1.0) | 1 (1.1) | 1 (1.1) | / | 2 (2.0) | 1 (1.1) | 1 (1.1) | / |
| GA (%) | 22.5 ± 4.1 | 17.2 ± 2.9 | 15.7 ± 2.0 | 15.3 ± 2.1 | 21.9 ± 3.5 | 17.0 ± 2.7 | 16.0 ± 2.5 | 15.7 ± 2.3 |
| Change of GA from baseline | / | -4.7 (-7.7, -3.0) | -5.9 (-9.7, -4.0) | -6.5 (-10, -3.8) | / | -4.5 (-5.8, -3.1) | -5.1 (-7.5, -3.8) | -5.6 (-8.0, -4.0) |
| HbA1c (%) | 8.9 ± 0.9 | / | / | 6.6 ± 0.6 | 8.7 ± 0.8 | / | / | 6.8 ± 0.6 |
| Change of HbA1c from baseline | / | / | / | -2.26± 1.03 | / | / | / | -1.93± 0.96 |

**Figure S1**：Study design

**Figure S2:** Enrollment, randomization and follow up of study participants

**Figure S3:** Changes in the proportion of monotherapy and combination therapy during follow up (A: GA guided therapy group; B: Standard therapy group)

**Figure S4:** Changes in GA during follow up

**Figure S5:** The changes in metabolic paramaters between the two groups (A: The change in body weight in FAS; B: The change in body weight in PPS; C: The change in BMI in FAS; D: The change in BMI in PPS; E: The change in waist circumference in FAS; F: The change in waist circumference in PPS)
